# Growth and Development of Preschool Children (12–60 Months): A Review of the Effect of Dairy Intake

**DOI:** 10.3390/nu12113556

**Published:** 2020-11-20

**Authors:** David C. Clark, Christopher J. Cifelli, Matthew A. Pikosky

**Affiliations:** 1 Bovina Mountain Consulting LLC, Englewood, FL 34223, USA; david@bovinamountain.com; 2 National Dairy Council, Rosemont, IL 60018, USA; Chris.Cifelli@dairy.org

**Keywords:** preschool children, milk intake, dairy intake, growth, development, weight, obesity

## Abstract

Undernutrition in young children is a global health issue. The ability to meet energy and nutrient needs during this critical stage of development is necessary, not only to achieve physical and mental potential but also socio-economic achievement later in life. Given ongoing discussions regarding optimization of dietary patterns to support achievement of the Sustainable Development Goals established by the United Nations, it is important to identify foods/food groups that have shown efficacy in reducing the negative impacts of undernutrition in young children. This narrative review addresses the impact of dairy intake, with a focus on linear growth, cognitive development and weight gain in early childhood (12–60 months). The impact of country economic status is also examined, to help elucidate regional specific recommendations and/or future research needs. Overall, the body of research addressing this age group is somewhat limited. Based on the data available, there is a positive association between dairy intake and linear growth. The impact of milk or dairy products on cognitive development is less clear due to a lack of evidence and is a gap in the literature that should be addressed. Regarding the impact on body weight, the majority of evidence suggests there is either no association or an inverse association between milk intake by preschool children on overweight and obesity later in life. This evidence is exclusively in high income countries, however, so additional work in lower income countries may be warranted.

## 1. Introduction

Undernutrition in young children is a global health issue. In the 2019 edition of the joint malnutrition estimates, the United Nations Children Fund (UNICEF) reported that nearly 149 million children under 5 years of age were stunted in 2018 [1]. Stunting is a global issue but is more concentrated in developing countries in Africa and Asia where, for example, Burundi, Eritrea and Timor-Leste have levels of incidence exceeding 50% in ages up to 60 months. In contrast, the last available data from the United States for year 2012 reported levels of 2.1% amongst children up to 5 years old [2]. Globally, over 49 million of the same age group suffered from wasting—i.e., low weight for height [1]. While the physical impact of stunting is evident, it is also associated with impairment of cognitive development and socio-economic potential. These developmental shortfalls resulting from undernutrition during this critical period are, for the most part, irreversible. Stunted children cannot recover height in the way they can regain weight following undernutrition. In addition, stunted children are more prone to sickness, often due to recurring infections, miss more lessons and underperform at school and ultimately are more likely to be economically disadvantaged [3].

Prevention of stunting frequently relies on nutritional intervention. However, evidence is emerging that it is not simply a matter of correcting a deficit in a single macro- or micronutrient but rather the delivery of a balanced diet with respect to energy intake and the ability of that diet to provide essential amino acids and minerals, such as zinc, potassium, sodium and phosphorus, for which there are no identified stores in the body [4]. Further, it is also not simply a matter of meeting a certain intake of protein but ensuring that the protein consumed is high quality and contributes a balanced source of essential amino acids [5]. In particular, the importance of incorporating animal sources of protein has been proposed, and milk and dairy are of specific interest as they deliver energy, high quality protein and a variety of micronutrients [6]. 

In this narrative review, literature that addresses the impact of dairy intake on undernutrition in early childhood (preschool years, 12–60 months), in high and upper-middle income countries is examined. Dietary changes in these countries include a shift towards highly processed foods as well as a recommendation, by some, towards substitution of plant in place of animal sourced protein/foods. Both changes have resulted in a decreased intake of dairy foods and macro- and micronutrients contained therein, which may negatively impact the nutritional status and development of young, preschool children. These findings are contrasted with data from the same age group of young children in low and lower-middle income countries. Finally, knowledge and research gaps are identified and discussed.

## 2. Methods

A literature search was conducted using PubMed, Google Scholar and Medline databases using combinations of key words including “young children” or “preschool children” and “milk intake” or “dairy intake” and “growth” or “development” or “weight” or “obesity”. The titles and abstracts of papers were screened, and relevant papers were assembled in a working database and classified by study type, primary research field (e.g., observational, linear growth) and economic status of the country in which the study was located. Copies of full text versions of these shortlisted papers were again reviewed; new citations were checked and out of scope papers (e.g., outside age range of 12–60 months) were rejected from the working database. In this narrative review, we focus on a subset of papers from the database that address dietary intake of “non-fortified” milk and dairy by preschoolers (12–60 months age group) and to what extent there exists evidence of an association with linear growth (height), cognitive development and weight gain in these subjects in countries of different income classification.

## 3. Impact of Undernutrition on Cognitive Development in Early Childhood 

It is estimated that at least 200 million children living in developing countries fail to meet their development potential [7,8]. Brain development in young children follows a continuum with different neural regions and processes peaking at different times. Cusick and Georgieff [7] reported that critical nutrients required during this developmental stage, include protein, long chain polyunsaturated fats, iron, copper, zinc, choline and vitamins, with protein, iron and zinc being common to all development stages. These authors also pointed out that brain development extends beyond the first one thousand days (i.e., conception to 2 years of age) to at least three years of age. Provision of optimum nutrition to prevent alterations to brain structure or function is necessary to avoid irreversible long-term consequences. Children from 1 to 3 years of age are particularly vulnerable because they typically ingest a diet similar to that of their parents. Thus, they are susceptible to adoption of poor parental food habits and can have food insecurity that causes a sacrifice of food quality for food quantity.

## 4. Linear Growth and Socio-Economic Potential 

Improper physical development can reduce an individual’s capability to achieve their potential. In the six-stage life cycle model of Hoddinott et al. [9], genetic and environmental endowments are fashioned by four risk factors, the first of which is inadequate food intake. While developmental status is initially defined by the outcome from the first thousand days, that in turn defines the starting point for the “preschool ages” stage that follows and on through the remaining four stages of the model. Hoddinott [9] goes on to cite evidence of an association between height and outcomes in the labor market in developing countries. For example, Thomas and Strauss [10] reported that, in Brazil, a 1% increase in height was associated with a 2.4% increase in adult male earnings. Perhaps more surprisingly, it has also been reported that height is associated with income in developed countries. Persico et al. [11], drawing on studies from the U.S. and Great Britain, concluded that taller workers received a wage premium compared to shorter workers. Their study indicated that those who are short as teenagers have lower earnings, even if their heights “catch up” by adulthood. Case and Paxton [12] linked height and cognitive development and proposed that taller children from the United States and United Kingdom have higher average cognitive scores and used this to explain the premium that height contributes to earnings. They stated that taller children perform significantly better on cognitive tests starting at age 3. More recently Sudfeld et al. [13] performed a meta-analysis on studies addressing linear growth (height-for-age z score (HAZ)) and cognitive function among children in low to middle income countries (LMIC). While this meta-analysis included studies that spanned an age range that was broader than 12–-60 months of interest in this article, these authors did find that each unit increase in HAZ during the first 2 years of life was independently associated with a +0.24-standard deviation (SD) shift in concurrent cognitive ability and a +0.22-SD shift in cognition at 5 to 11 years of age.

Thus, there is mounting evidence supporting an association between height, cognitive ability and an individual’s socio-economic potential. Which aspects of nutrition significantly impact height and cognitive ability? A recent ecological study analyzed the main correlates of male height in 105 countries in Europe, Asia, North Africa, and Oceania [14]. Data on male height were compared with the average consumption of protein from 28 different sources and with 7 socioeconomic indicators. The most significant nutritional correlates of stature were intake of milk proteins (r = 0.79; *p* < 0.001), followed by total protein (r = 0.74; *p* < 0.001), and then animal protein (r = 0.73; *p* < 0.001). Taken together, a picture emerges showing an association between height and cognitive ability with achievement of potential and that an individual’s path is set at an early age.

## 5. Observations and Recommendations on Dairy Intake by Preschoolers

Milk and dairy products are an important source of essential nutrients required for normal growth and development. In the U.S., analysis of the National Health and Nutrition Examination Survey (NHANES) data from 2011–2014 showed that milk was the main source of three of the four “nutrients of public concern” in young children, identified by the 2015 Dietary Guidelines for Americans [15]; calcium, vitamin D and potassium [16]. Globally, the role of milk and dairy products in the diet are discussed in Chapter 4 of the Food and Agriculture Organization (FAO) report entitled *Milk and Dairy Products in Human Nutrition* [17]. The report states that a 250 mL glass of whole milk would supply a 5–6-year-old child with ~48% of protein requirements, 9% of calories and key micronutrients [18].

The scientific evidence supporting the benefits of milk consumption by young children was also recently acknowledged in a Consensus Statement published by a conglomerate of key national health and nutrition organizations in the United States [19]. Specifically, the panel of experts recommended consumption of 2–3 cups per day of whole milk for the age group 12–24 months and ~2 cups per day of nonfat/lowfat milk for 2–5-year-olds. The report also noted that plant-based milk alternatives (except for fortified soy beverages), flavored and toddler milks, sugar sweetened beverages, and beverages containing low calorie sweeteners or caffeinated beverages should be avoided (Table 1). Unfortunately, recommendations are not consistent across global organizations, which can lead to confusion. For example, the World Food Programme (WFP) also recently published a position paper entitled *Use of Milk in WFP Operations* [20]. The WFP recommends restricting the use of milk in rations for children to only those above the age of 2 years. The argument used by WFP for excluding milk in the diet of toddlers relates to concerns about it being employed as a breastmilk substitute, thus leading to the shortening of breastfeeding to a period less than their recommended 24 months. In contrast, leading authorities in the U.S. recommend exclusive breastfeeding until 6 months [21]. Should the mother choose to wean their infant at age 6–12 months, mothers are advised to supplement weaning foods with formula. If weaning occurs after 12 months of age, it is recommended that the toddler is transitioned directly to 2–3 cups of whole cow’s milk per day until they reach 24 months followed by ~2 cups per day of non or low-fat milk for 2–5-year-olds [19].

## 6. Dairy Intake and Linear Growth in Preschool Children

A quarter of the original research studies cited in this section addressing the association of dairy intake on linear growth (Table 2) were performed in LMIC (5 out of 19). For the purposes of this review, we excluded studies that only compared fortified milk against a non-fortified control milk. Seventeen of the nineteen studies reviewed reported a positive association between non-fortified (i.e., milk that was not fortified by added micronutrients other than Vitamin D) milk intake and linear growth. The two studies which reported no association or insufficient data to make a judgement were performed in high-income countries [22,23]. 

Given the critical nature of ensuring nutrient adequacy during early childhood, there are surprisingly few studies that specifically address this age group [41]. Indeed, meta-analyses and systematic reviews addressing possible associations between dairy intake and linear growth generally cover a broader age range, combining analysis of data from studies of preschoolers with those of older children and teenagers. For example, the meta-analysis of de Beer [42] reviewed data from 12 studies, which included only one study within the 12–60 month age group [34]. In He [34], it was reported that a 9-month intervention with 125 mL yogurt per day resulted in a 0.02 cm height increase per month compared to the controls (Table 2). This same study [34] was the only age-relevant study that met inclusion criteria in a more recent meta-analysis [43] of 13 dietary intervention studies involving dairy intake and height. De Lamas [43] reported that intervention with yogurt showed no significant difference when compared to studies using milk. These authors also reviewed bone mineralization studies, but none fell within the 12–60-month age range relevant to this paper.

Observational research indicates that preschool children who consume more cow’s milk are taller than those who drink less [33,35,36] (Table 2). The first study of U.S. preschool children to show a positive association between This milk consumption and height among young children (age 24–59 months) was that of Wiley [35]. Using NHANES 1999–2004 data, they reported that those in the highest quartile of milk intake (average = 502 g milk in 24-h recall) had greater height percentiles than those in each of the lower quartiles. Similarly, DeBoer et al. [37] found that greater milk consumption was associated with a higher height z-score at ages 4 and 5 among children in the Early Childhood Longitudinal Study who drank milk at age 4 (Table 2). Finally, Hoppe et al. [33], found positive correlations between height and milk consumption in a sample of 2.5-year-old Danish children (Table 2). Specifically, each 100 g increment of additional milk intake corresponded to 0.5 cm greater height in multivariate regression models.

A New Zealand study [31] of a broader age group (3–10-year-olds) demonstrated that those who avoided cow’s milk were shorter than those who consumed milk (Table 2). However, it should be noted that the researchers did not control for differences in energy intake in this study. A further confounder was that half of the children who avoided milk had some form of intolerance, including cow milk allergy, and most reported other atopic issues as well. Thus, it is possible that lower overall dietary intake and/or steroidal treatment for atopy might contribute to the observed outcome.

Dror published two reviews on linear growth, one which addressed the association of dairy intake with linear growth and bone health in developed countries [44]. Seventeen observational and intervention studies were reviewed but only four are relevant to this review from an age perspective. Three of the four studies showed a significant positive association between milk consumption and height [31,33,35] (Table 2). The remaining study [22] reported no difference in height at 8 years old by quintile of dairy intake at 1.5 years age. Dror [44] concluded that dairy products are important for linear growth during childhood in developed countries. This finding broadly agreed with the outcomes of the meta-analysis of de Beer [42], comprising studies from both high- and low-income countries. The latter showed that the most likely effect of dairy products supplementation is 0.4 cm per annum additional growth per approximately 245 mL daily intake of milk. However, as noted above, the de Beer meta-analysis relies almost exclusively on data from studies on subjects older than the 12–60 months age range addressed in this paper.

The literature review of Hoppe [45]—comparing observational and intervention studies in developing countries with those from developed countries, including a series of studies relating to the work of Martorell [26,27,28] (Table 2) that compared consumption of a nutritional drink (Atole), comprising milk powder and cereal with a fruit drink by preschool age Guatemalan children—is most relevant to this review. Hoppe [34] concluded that it was not clear whether the effects of the Atole on height were due only to its energy content or the nutrient contributions from dried skim milk and cereal in this supplement. Hoppe [45] also cited the article of Ruel [32], who analyzed the association between height and intake of milk, meat, and egg/fish/poultry products in the Demographic and Health Survey. Milk intake was found to be significantly associated with higher height-for-age z-scores in all seven Latin American countries, whereas meat and egg/fish/poultry intakes were only associated with height in one of the countries. Hoppe also reported that Allen [30] found positive associations between the intake of specific foods and linear growth of Mexican toddlers. The typical diet of taller toddlers contained more animal products, especially milk and meat, than that of shorter children. Finally, Hoppe [45] also described results from their own study, which was conducted amongst well-nourished Danish preschoolers. This study showed that height was positively associated with intake of animal protein and milk but not vegetable or meat protein [33].

The current literature search identified multiple randomized, controlled trials that evaluated the effect of the consumption of dairy foods, fortified dairy foods, or supplemental dairy foods on linear growth. Four out of a total of 5 studies showed a statistically significant association between milk intake and growth [23,24,25,29,38]. The studies were conducted in countries covering a variety of levels of economic status with high, upper and lower-middle income represented (Table 2). Of this group, the study that did not deliver a statistically significant association was that of Elwood [23], which relied on the use of tokens to obtain milk at a discounted price. The lack of an effect might be due to poor compliance, which was reported at only 40%. Further evidence supporting the positive effect reported from the randomized controlled studies is provided by the very recently published cross-sectional study of Duan et al. which reported that dairy intake was significantly associated with a higher height-for-age Z-score (HAZ) and lower risk of stunting for children aged 2–4 years in China [40]. The risk of stunting for children who consumed dairy at least once per day was 28% lower than the children without dairy intake in the last week, and the risk was similar between weekly dairy consumption and no dairy consumption (AOR: 1.03, 95% CI: 0.74–1.42) after adjusting for potential confounders, including socioeconomic characteristics, lifestyle, health status, and the intake frequency of other foods.

It is notable that the outcomes of some studies have not identified a positive association between dairy intake and linear growth. For example, a systematic review from Hornell et al. [46] concluded that there was only limited suggestive evidence that the intake of animal protein, especially from dairy, has a stronger association with growth than vegetable protein. Further, a recently published longitudinal study by Marshall et al. [39] reported that milk intake adjusted for mean adequacy ratio, energy intake, and baseline socioeconomic status was associated with height throughout childhood and teenage years. However, a common factor shared by these reports is that they include data from a much broader age range than is addressed in our review and this may have had an impact on outcomes.

In summary, data from studies that focus on preschool or young children suggest that there is a positive association between dairy and milk intake and increased linear growth.

## 7. Cow’s Milk Exclusion Diets

Further evidence that indirectly supports an association between dairy intake and linear growth during preschool years comes from studies that examined the impact of diets that excluded cow’s milk. Subjects on cow’s milk exclusion diets fall into two categories. First, therapeutic milk exclusion diets are frequently applied in subjects displaying allergic responses to milk protein (cow’s milk allergy (CMA)). Second, are those whose parents/guardians for ethical or religious reasons feed their children diets containing little or no animal products.

Evidence indicates that the growth of children with food allergies on an elimination diet can be compromised. Milk exclusion diets eliminate a good/excellent source of a wide range of nutrients that are difficult to replace without careful dietary planning. Henriksen et al. [47] concluded that children aged 31–37 months consuming cow’s milk-free diets had increased risk of malnutrition. They stated that exclusion of milk may contribute to the observed growth retardation seen among children with adverse reactions to cow’s milk consumption.

The growth of children on exclusion diets was reviewed by Mehta et al. [48]. Some of the studies discussed by Mehta involved rather small numbers of subjects. For example, Tiainen et al. [49] studied 18 children with challenge-proven cow’s milk allergy compared to 20 healthy children (mean age 2.0 years). While there was no difference in energy intake between the groups, protein intake by the allergic children was lower (39 g vs. 48 g) and fat intake higher (47 g vs. 39 g) than that of the healthy children. In addition, the height-for-age was lower in the children with cow’s milk allergy (−0.6 vs. +0.2 SD) compared to healthy children.

A larger cohort of 90 children on cow’s milk exclusion diets was studied by Tuokkola et al. [38]. Weights and heights were recorded up to 5 years of age. Children on the cow’s milk elimination diet for at least one year were slightly smaller than expected. When examined longitudinally, the growth of children on the milk elimination diet decelerated within the first year on the diet, after which the difference of 0.2–0.3 SD between the cases and controls remained the same. Importantly, no catch-up growth was seen by the age of five years.

There is evidence that failure in catch-up growth is an irreversible phenomenon. Recently, a study of young Israeli adults (average age 19.5 years) who had CMA from infancy and followed cow’s milk elimination diets showed an increased risk of not reaching their growth potential [50]. Mean values of height z-scores were significantly reduced in CMA subjects compared with controls. Patients with CMA had significantly lower intake of protein, and several essential vitamins (A, B12, and riboflavin) and minerals (calcium, potassium, phosphorus, magnesium, and zinc) compared with controls. This observation may be due to an inability to adequately compensate for elimination of milk and dairy products from the diet.

Concerns about the risk of nutritional deficiencies in young children fed “plant-milks” as substitutes for cow’s milk first drew attention almost 20 years ago. For instance, increased kwashiorkor and rickets were observed with increased plan-milk consumption by Carvalho et al. [51]. More recently, Morency et al. [52] examined plant-milk intake and height-for-age in 5034 healthy Canadian children aged 24–72 months. There was a dose-dependent association between higher plant-milk consumption and lower height. For each daily cup of plant-milk consumed, children were 0.4 cm (95% CI: 0.2, 0.8 cm) shorter. In the mediation analysis, lower cow’s milk consumption only partially addressed the association between plant-milk consumption and lower height. The height difference for a child aged 3 years consuming 3 cups of plant-milk/d relative to 3 cups of cow’s milk/d was 1.5 cm (95% CI: 0.8, 2.0 cm). Additional evidence comes from the study of Headey and colleagues [53], who found that children aged 6–23 months with vegan mothers were 5.2% more likely to be stunted when compared to those with non-vegetarian mothers in a cross-sectional study from India. However, associations for other age ranges were not statistically significant in their study.

In comparing the nutrition impact of cow’s milk and plant-milks, it is important to note that there are no regulatory requirements for standardizing the nutritional content of plant-milk under the Food and Drug Administration (FDA) or Codex Alimentarius. Accordingly, the protein and fat content of plant-milk beverages can be highly variable. In addition, fortification of these beverages with micronutrients is inconsistent as some are fortified with select micronutrients, such as calcium, vitamins A and D, while others are not fortified at all. Children who consume plant-milk may receive less dietary protein and fat than children who consume cow’s milk. The recent Consensus Statement on Healthy Beverage Consumption in Early Childhood recommends that, with the exception of fortified soy milk, plant milks are not nutritionally equivalent to cow’s milk and should only consumed by young children in cases where there are medical indications or special dietary reasons [19]. Additionally, the US Dietary Guidelines state only fortified soy beverage can be used as a substitute for dairy milk. Other products sold as “milk” but made from plants (i.e., almond, rice, coconut and hemp) are not included as part of the dairy group because their overall nutritional content is not equivalent to dairy milk and fortified soy beverages [15].

Cow’s milk allergy is displayed by 2–6% of infants. While most infants outgrow the condition within 1–3 years, it is critical that children receive the macro- and micronutrients needed to ensure normal growth and development. Indeed, the research summarized above show young children who avoid cow’s milk may suffer from reduced linear growth. Furthermore, the evidence presented above is strongly suggestive that those deficits in linear growth resulting from cow’s milk exclusion in preschool years will likely not be recovered even when consumption of cow’s milk is resumed. Thus, ensuring proper education and nutritional advice for those on cow milk exclusion diets is essential, as the nutrients found in milk are not easy to replace.

## 8. Dairy Intake and Cognitive Development in Preschoolers

Only a few studies have examined the link between dairy intake and cognitive development. The majority of the studies that examine diet and cognitive development in children do not typically differentiate between dairy and other animal sources of protein [26]. Frequently, studies that do extract dairy intake data examine the potential beneficial effect of iron-fortified dairy products rather than unadulterated milk or dairy products (for review, see Grantham-McGregor [54]). One exception is the study by Grantham-McGregor [55], which involved the supplementation of Jamaican children aged 9–24 months with a milk-based formula for 2 years and follow-ups with the subjects at 7–8 years [56] and again at 18 years [57]. The results showed an association between stunting in early childhood and cognitive and educational deficits in late adolescence [57]. Interestingly, these effects were reduced by both supplementation with milk-based formula and cognitive stimulation at a young age. Abdel-Rahman [58] reported on nutritional status and cognitive development amongst preschool children in Egypt. The cohort was comparatively well-nourished with only 9.4% stunted, although a further 28.8% were considered at risk of stunting. The study showed that the children of families cooking with margarine rather than dairy fat were more likely to have a lower IQ score.

Taken together, results on association between dairy intake and cognitive function/development are too limited to reach a conclusion. In many cases, there is no specification of which source of animal protein was reported as consumed (e.g., meat vs. dairy) and the age ranges investigated in most studies were much broader and older than the 12–60 month focus of this paper. There is a clear need for more studies in this important area of nutrition and development particularly addressing preschool age children.

## 9. Dairy Intake and Weight Gain

Several publications have reported associations between dairy intake during infancy and weight gain—for example, Weber et al. [59] and findings have been used to fine tune regulations governing infant formula composition. However, for the purposes of this review, the focus is on the link between dairy intake during the preschool years (12–60 months) and weight gain. Assessment of whether dairy intake by the preschool age group is associated with weight gain is very important, since dairy products frequently comprise the main source of protein and saturated fat in the diet of these young children. As a result, increased dairy intake during childhood has been scrutinized with respect to its effect on weight, body composition and risk of overweight, obesity and other non-communicable diseases of later life. Papers reporting original research on dairy intake and risk of overweight and obesity are summarized in Table 3.

Hornell [46] systematically reviewed protein intake as part of the 5th revision of the Nordic Nutrition Recommendations, but across an age range of 0–18 years. Of the 23 papers in their review that addressed protein intake and body mass index (BMI), growth or body composition, only 8 fell substantially within the 12–60 month age range. The general conclusions from the review were that protein intake between 15 to 20% of energy in early childhood was associated with an increased risk of being overweight later in life. However, there was only limited-suggestive evidence that intake of animal protein, especially from dairy, had a stronger association with growth than vegetable protein. Only three of the age-relevant papers reviewed specifically addressed dairy intake (Table 3). Hoppe et al. [33] found an association between milk intake and serum IGF-1 levels and height in 2.5-year-old boys. Gunther et al. [60] reported that the percentage of energy from dairy protein at 12 months, but not meat or cereal protein, was not positively associated with BMI at 7 years. Finally, Ohlund et al. [61] reported that the intake of protein and energy from milk at 4 years was positively associated with BMI z-score at 4 years.

The above papers investigated associations between dairy-derived protein and/or energy intake and body weight. Other papers have examined the impact of simple dairy intake and metrics of weight. For example, DeBoer [37] found that higher milk consumption was associated with higher z-scores of BMI as well as height and weight-for-height in children 4 years of age. This corresponded to differences of approximately 0.15 kg in weight between children drinking <1 and ≥4 servings of milk daily. However, this association was no longer present by age 5 years. At 4 years, children drinking ≥3 servings of milk daily were more likely to be overweight/obese (BMI ≥ 85th percentile) than those drinking 0.5–2 servings of milk daily.

Other studies focused on comparing consumption of different beverage types or food groups by young children. Dennison [62] reported that the prevalence of obesity was not increased among children drinking ≥ 16 fluid (fl) oz/day of milk compared with those drinking less than 16 fl oz/day of milk. A recently published systematic review and meta-regression analyses from Garrido-Miguel et al. [63] found the consumption of animal proteins, and sugars was positively associated with excess weight, whereas the consumption of milk and dairy products was inversely associated with overweight and obesity among European preschool children, indicating that the source of protein should be considered when examining this question vs. simply grouping animal proteins together collectively. Similarly, a meta-analysis by Dror and colleagues showed no association between dairy intake and adiposity [64] among children in the preschool and school age ranges. However, there was a modestly protective effect in adolescence. Another meta-analysis by Lu [65] evaluated dairy consumption and risk of overweight/obesity using data pooled from four prospective cohort studies, three of which fell within the age range of interest in this review and comprised, in total, 20,050 subjects [65,66,67] (Table 3). The study of Scharf [66] showed that children in the highest intake group of dairy were 36% less likely to have overweight/obesity as compared with those who were in the lowest intake. The study of Huus [67] examined associations between dairy product type and showed a reduced probability of childhood overweight/obesity when milk or cream/crème fraiche were consumed but not cheese or ice cream. Finally, the study by Huh [68] focused on the fat content of milk but showed no clear difference between whole milk, reduced fat, or 1%/skim low-fat milk.

The only age-relevant study in the meta-analysis by Lu [65] that examined BMI was conducted by Moore [69]. This study showed that dairy product consumption was linked to a reduction in BMI in the pooled data. Finally, Carruth and Skinner [70], showed an association between dairy product consumption and lower percentage of body fat. They concluded that higher longitudinal intakes of calcium, monounsaturated fat, and servings of dairy products were associated with lower body fat. The review by Abreu [71] also captured most of the above studies along with that of Newby [72], who reported weight change and BMI were not significantly related to milk intake in 2–5-year-olds.

A number of studies have compared the associations between non-/low-fat milk, reduced-fat (2%) and whole milk with overweight and obesity. Scharf [66] reported that preschool children consuming 1%/non-fat milk at age 2 years had higher odds of overweight and obesity at ages 2 and 4 years, as compared to those consuming 2% or whole milk. Although change in BMI or BMI z-score between time points was not predicted by the fat content of milk, normal weight children who consistently consumed 1%/non-fat milk had significantly higher odds of overweight or obesity by age 4 years.

The literature review of Abreu [71] included three cross-sectional and six longitudinal publications that were age-relevant. The study of O’Connor [73] examined NHANES data from 1999–2002 and found no association between types of milk (skim, 1%, 2%, whole and flavored) and BMI in 2–5-year-olds. Similarly, La Rowe [74] observed no association in the 2–5-year age group but did find that BMI was significantly lower amongst high fat milk consumers in the 6–11-year age group. Only Wiley [75] reported that young children aged 2–4 years in the highest quartile of dairy and milk intake had higher BMIs than those in the lowest quartile. However, adjustment of the data for energy intake eliminated these effects [75].

Vanderhout [76] identified that there was an inverse association between milk-fat percentage and BMI z-score irrespective of amount consumed among the 2745 children included in their Canada-based study. Beck [77] collected data on 145 Latino 3-year-olds living in California of whom 17% were severely obese. Severely obese children had a lower mean intake of milk fat (5.3 g vs. 8.9 g) and fewer drank any milk (79% versus 95% for not severely obese children). Indeed, higher milk fat consumption was associated with lower odds of severe obesity (OR 0.88 CI 0.80–0.97).

In summary, the preponderance of the evidence suggests that there is no association (7 of 16 papers in Table 3) or an inverse association (5 of 16 papers in Table 3) between intake milk and overweight or obesity in young children aged 12–60 months. In most of these studies, milk consumption showed no association or in some cases an inverse association with BMI or obesity. However, in studies where total protein intake was between 15 and 20% of energy in early childhood, milk intake was associated with increased risk of overweight.

It is important to note that almost of the studies reviewed were conducted in children living in high income countries. Thus, additional research is needed to more fully understand the link between dairy foods and dairy protein on weight in 12–60-month children from LMIC.

## 10. Discussion

Associations between diet and linear growth, cognitive development and socio-economic potential of individuals have been previously reported. Linear growth and cognitive development are particularly influenced by nutritional status in young children and deficits in nutrition during preschool years may be irreversible, resulting in failure to achieve not only physical stature but also socio-economic potential. Thus, as noted in this review, it is surprising that the body of nutrition research specifically addressing the preschool age group of 12–60 months is comparatively limited [41].

Milk and dairy products constitute a significant part of the diet of preschool age children in high income countries, where dairy provides a rich source of protein, fat, calcium and vitamin D. Given this prominent position in the diet of young children, there have been markedly few studies that examine possible associations between milk and dairy product intake and achievement of linear growth and cognitive potential. Within this comparatively small body of work, studies that address associations between dairy intake and linear growth in children 12–60 months consistently showed a positive association between dairy intake and linear growth. Half of the studies examined, including the two studies which reported no or insufficient data to make a determination of existence of an association, were performed in high income countries (Table 2).

Aside from gaps in knowledge resulting from so few age-relevant milk intake studies in lower income countries, the causative component(s) responsible for the association between milk/dairy consumption and height are as yet undefined. Some articles implicate milk protein, others suggest components in milk stimulate IGF-I secretion, or the possible involvement of unique bioactive compounds present in milk and dairy could be directly impacting linear growth. This could be an area for future research. The data also show that when milk is eliminated from the diet of preschool children due to cow’s milk allergy or complete substitution with plant-milk products due to parental preference, an association with a reduction in height is observed that appears to be irreversible.

The consumption of a diet adequate in milk or dairy products and cognitive development is far less clear due to the dearth of studies. While some studies have been conducted in lower income countries, most of these studies have compared fortified milk with controls comprised of non-fortified milk. A clearer understanding will only emerge when studies that also include a non-dairy control arm are conducted. Additionally, research needs to examine how milk intake could impact iron intakes, especially in children 12–24 months and in lower income countries. Iron is essential for normal cognitive development and high intakes of milk or calcium could negatively impact iron status because calcium and iron compete for the same uptake mechanism. While milk provides numerous essential nutrients needed for optimal growth, dietary balance is needed to ensure that children receive all of the nutrients they require. Finally, future work should look at the impact of specific dietary patterns, such as the Mediterranean diet, on linear and cognitive growth in this population to help inform future dietary recommendations. A recently published Consensus Statement on Healthy Beverage Consumption in Early Childhood [19] recommended that 12–24 month old toddlers consume 2 cups of whole milk and children aged 2–5 years should consume 2–2.5 cups of non/lowfat milk per day. This caution about the fat content of the recommended milk type derives from concerns about the potential for full-fat dairy intake in young children to lead to overweight or obesity later life. The current evidence suggests that there is no association between milk intake during preschool years with increasing BMI. Indeed, the balance of evidence would suggest an inverse association with overweight or obesity, regardless of the fat level of the milk. However, these observations may have been influenced by other confounding factors that should be better accounted for in future studies. Additionally, the absence of data from studies conducted in low- and middle-income countries is a serious gap, especially given the dual burden of undernutrition and overnutrition that is becoming prevalent in developing countries.

Finally, it is notable that most of the studies that address both linear growth and overweight/obesity and diet examined diet only by food group. Thus, milk or dairy intake was not broken down by dairy product type such as fluid milk, yogurt or cheese or for that matter milk fractions, such as milk or whey protein concentrates. As a result, it is an open question whether milk proteins or a larger part of the milk complex is responsible for supporting linear growth in young children. The addition of this level of detail to future studies would generate insights into whether associations were a function of the total milk matrix or a specific dairy product type.

## Figures and Tables

**Table 1:** Summary of key panel findings—reproduced from Healthy Eating Research, 2019 [19].

Beverage Type	0–6 Months	6–12 Months	12–24 Months	2–3 Years	4–5 Years
Plain Drinking Water	Not needed	0.5–1 cups/day	1–4 cups/day	1–4 cups/day	1.5–5 cups/day
Plain Pasteurized Milk	Not recommended		2–3 cups/day whole milk	≤2 cups/day skim or low-fat milk	≤2.5 cups/day skim or low-fat milk
100% Juice	Not recommended		≤0.5 cups/day		≤0.5–0.75 cups/day
Plant milks/Non-dairy beverages	Not recommended		Medical indication/dietary reasons only		
Flavored milk	Not recommended				
Toddler Milk	Not recommended				
Sugar-sweetened beverages	Not recommended				
Beverages with low-calorie sweetener (LCS)	Not recommended				
Caffeinated beverages	Not recommended				

**Table 2:** Original observational and intervention studies of dairy intake and height change in children aged 12–60 months.

Reference	Location (Income Category) ^1^	Study *N* ^2^	Age (year)	Design	Methods	Outcome on Height	Results
**Randomized Controlled Studies**							
Super (1980) [24]	Colombia (Upper-Middle Income Country (UMIC))	N/A	Up to 5	Randomized Controlled	Supplement with 60 g Skim Milk powder (SMP) at (a) pregnancy to 6 m; (b) 3–36 m; (c) a + b	Positive	At 3 years, children who had received food supplementation averaged 2.6 cm and 642 g larger than controls
Elwood (1981) [23]	United Kingdom (High Income Country (HIC))	510	Up to 5	Randomized Controlled	Tokens supplied to treated allowing purchase of one pint of milk at 50% cost	Not significant	Compliance was poor. Only 40% of provided tokens were utilized
Walker (1991) [25]	Jamaica (UMIC)	64	0.75–2	Randomized controlled	One kg milk-based formula per week delivering 20 g protein per day	Positive	After 12 months, supplemented children had significantly increased length, weight, and head circumference
**Observational Studies**							
Martorell (1979) [26]	Guatemala (Lower-Middle Income Country (LMIC))	125–178	1.25–3	Cohort extracted from Longitudinal	Daily intake of supplement (Atole) and control (Fresco) measured to nearest 10 mL	Positive	Protein-calorie intake was strongly related to growth in supine length. Effect may have been due to non-iso-energetic test material
Martorell (1980a) [27]	Guatemala (LMIC)	190–327	0.25–7	Cohort extracted from Longitudinal	Daily intake of supplement (Atole) and control (Fresco) measured to nearest 10mL	Positive	Growth rates at 2–3 years of age were most affected. For 3–7 years, the impact supplements on growth rates was quite small. Effect may have been due to non-energy balanced test and control materials
Martorell (1980b) [28]	Guatemala (LMIC)	229	0–3	Cohort extracted from Longitudinal	Daily intake of supplement (Atole) and control (Fresco) measured to nearest 10 mL	Positive	A statistically significant increase in supine length was observed in the group receiving the test material containing dried skim milk. Effect may have been due to non-energy balanced test and control materials.
Vaughan (1981) [29]	Sudan (LMIC)	287	0.5–2.2	Cohort with control	Fortnightly provision of 1 kg SMP or local beans	Positive	40% of children showed an improvement in weight-for-age and weight-for-height categories. SMP supplemented group grew on average 0.24 cm per month more than those in the beans group
Allen (1992) [30]	Mexico (UMIC)	64	1.5–2.5	Longitudinal	Maternal recall, observation weighing and food record, 2 adjacent days per month.	Positive	Size at 30 months and growth rates were positively related to consumption of animal-origin foods.
Black (2002) [31]	New Zealand (HIC)	250	3–10	Cross-sectional	Food Frequency Questionnaire (FFQ)	Positive	Milk avoiders were significantly shorter than control children of the same age and sex
Ruel (2003) [32]	5 Latin American countries (UMIC/LMIC)	N/A	1–3	Cross-sectional	7 data sets from Demographic and Health Surveys	Positive	Milk intake was associated with higher height-for-age Z-scores (HAZ) and the effect was independent of breastfeeding status despite wide variations in milk intake by country.
Hoppe (2004) [33]	Denmark (HIC)	90	2.5	Cross-sectional	7-days food record	Positive	Milk significantly associated with height
He (2005) [34]	China (UMIC)	201	3–5	Cohort study	1 serving of yogurt (125 g) 5 day/week	Positive	Height gain in yogurt group was significant compared to control after 3, 6 and 9 months
Wiley (2009) [35]	United States (HIC)	1002	2–4	Cross-sectional (NHANES 1999-2002)	24-h recall, ranked milk consumption in past 30 days	Positive	Children in highest milk quartile (Q-IV) sig taller than those in Q-II and Q-III but not in Q-I. Children w/ daily milk intake were sig taller than those with less frequent intake. Other dairy not associated w/ height.
Wiley (2011) [36]	United States (HIC)	201	<5	Cross-sectional (NHANES 1999-2004)	(NHANES) data from 1999 to 2004	Positive	Milk intake and linear growth in early childhood and adolescence, but not middle childhood, a period of relatively slow growth
Rangan (2012) [22]	Australia (HIC)	335	1.5	Prospective cohort	3-d weighed food record	No change	No difference in height at 8 years by quintile of dairy consumption at 1.5 y
DeBoer (2015) [37]	United States (HIC)	8950	4–5	Longitudinal	Parental interviews including type and frequency of beverage intake	Positive	At 4, higher milk consumption was associated with higher z-scores for height). This corresponded to differences between children drinking <1 and ≥4 milk servings daily of 1cm in height
Tuokkola (2017) [38]	Finland (HIC)	90	0–3	Case Control	3-d food record at 1, 2 and 3 years	Positive	Children on milk elimination diets grew slower than controls
Marshall (2018) [39]	United States (HIC)	717	2–17	Longitudinal	Beverage intakes (*n* = 708) were collected by beverage frequency questionnaires at 3- to 6-m intervals	Positive	For each additional 8 ounces (236 mL) of milk consumed per day throughout childhood and adolescence, height increased, on average, by 0.39 cm
Duan (2020) [40]	China (UMIC)	12,153	2–4	Cross-Sectional (CNHS 2013)	Food Frequency Questionnaire (FFQ)	Positive	Dairy intake was significantly associated with higher HAZ and lower risk of stunting. Children who consumed dairy intake at least once per day had a 28% lower risk of stunting compared to children without dairy intake in the last week.

^1^ Income category refers to classification according to World Bank 2017; ^2^ *N* = subjects treated/receiving intervention, where controls were included; N/A = Not applicable.

**Table 3:** Original observational and intervention studies of dairy intake and weight in children aged 12–60 months.

Reference	Location (Income Cat.) ^1^	Study *N* ^2^	Age (year)	Design	Methods	Outcome on Weight	Results
Dennison (1997) [62]	USA (HIC)	90 and 70	2 and 5	Cross-sectional	Parents guided in completion of a written, consecutive, 7-day dietary record for their child	Not Significant	No association was observed between obesity and milk consumption. Prevalence of obesity was not increased among children drinking ≥16 fl. oz/day of milk compared with those drinking less than 16 fl. oz/day of milk
Carruth (2001) [70]	USA (HIC)	53	2–5	Longitudinal	18 days of dietary data collected 2–96 months (m) study	Inverse	Higher longitudinal intakes of calcium, monounsaturated fat, and servings of dairy products were associated with lower body fat.
Hoppe (2004) [33]	Denmark (HIC)	90	2.5	Longitudinal	7-day dietary record and serum IGF-I measurement	Not Significant	Weight was also positively correlated with total protein intake. However, the association with animal protein was not significant, whereas that with vegetable protein was significant
Newby (2004) [72]	USA (HIC)	1345	2–5	Longitudinal	Dietary and anthropometric data collected during WIc clinic visits on average 8.4 months apart	Not Significant	Weight change was not significantly related to intakes (per ounce) of milk
Moore (2006) [69]	USA (HIC)	92	3–6	Longitudinal	Dietary intake assessed repeatedly using 3-day diet records	Inverse	Suboptimal dairy intakes during preschool in this cohort were associated with greater gains in body fat throughout childhood. Both BMI and averages of 4 skinfold analyses showed significantly higher values for lower tertile of dairy intake compared to highest.
O’Connor (2006) [73]	USA (HIC)	1572	2–5	Cross-sectional	24-h dietary recall	Not significant	There was no clinically significant association between the types of milk (percentage of fat) consumed and weight status. There was not a statistically significant increase in BMI based on quantity of milk, 100% fruit juice, fruit drink, or soda consumed
Gunther (2007) [60]	Germany (HIC)	203	0.5–2	Longitudinal	The median of energy-adjusted protein intakes (in g/day) was used to distinguish different patterns of low and high protein intakes throughout the first 2 years of life	Positive in relation to dairy protein intake	Dairy but not meat or cereal protein intake, at 12 months was related to BF% at 7 years
LaRowe (2007) [74]	USA (HIC)	541	2–5 and 6–11	Cross-sectional	Diet quality was assessed using energy, micronutrient intakes, and Healthy Eating Index scores. Subjects clustered by beverage consumption	Not significant at preschool; inverse at school age.	Adjusted mean BMI not significant for preschool group but differed significantly across beverage clusters only in school-aged children but high fat milk cluster returned lowest BMI.
Huus (2009) [67]	Sweden (HIC)	16,058 to 7356	0–5	Longitudinal	Food frequencies reported by parents at 2.5 and 5 years were studied in relation to overweight/obesity at 5 years	Not significant	Intake of milk at 2.5 and 5 years showed no association with overweight/obesity. Intake of cheese at 2.5 years was positively associated with overweight/obesity at 5 years but only for the un-adjusted OR. At 5 years cream/creme fraiche were negatively associated with overweight/obesity.
Huh (2010) [68]	USA (HIC)	852	2–3	Cohort study	Milk and dairy intake at age 2 by semi-quantitative child food frequency questionnaire previously validated among preschool-age children	Not significant	Higher intake of whole milk at age 2, but not low-fat milk, was associated with a slightly lower BMI z-score at age 3. Intake of milk at age 2, whether full or low-fat, was not associated with risk of incident overweight at age 3. Neither total milk nor total dairy intake at age 2 was associated with BMI z-score or incident overweight at age 3.
Ohlund (2010) [61]	Sweden (HIC)	127	1.5–4	Longitudinal	Monthly 5-day food records during the initial 6–18 months of age and one 5-day record at the 4 years	Positive with respect to dairy protein and E% intake	BMI at 6–18 months was the strongest predictor of BMI at 4 years. Protein intake at 17–18 months and at 4 years, energy intake at 4 years and the father’s BMI were also independent contributing factors to the child’s BMI. There was a positive relationship between intake of protein and E% from milk at 4 years and BMI z-score at 4 years.
Wiley (2010) [75]	USA (HIC)	1493 and 2526	2–4 and 5–10	Cross-sectional	24-h recall and reported their past 30-day milk intake frequency	Positive association between E% from milk and BMI	Younger children in the highest quartile of dairy intake had higher BMIs than those in the lowest two quartiles in a non-energy adjusted model. Young children in the highest quartile of milk intake had higher BMIs than all lower quartiles
Scharf (2013) [66]	USA (HIC)	2745	1–6	Longitudinal	Parental computer-assisted interview including questions on fat-content of milk consumed, frequency and volume	Inverse association with fat content of milk	Increasing fat content in the type of milk consumed was inversely associated with BMI z-score. Compared to those drinking 2%/whole milk, 2- and 4-year-old children drinking 1%/skim milk had increased adjusted odds of being overweight) or obese. In longitudinal analysis, children drinking 1%/skim milk at both 2 and 4 years were more likely to become overweight/obese between these time points
DeBoer (2015) [37]	USA (HIC)	8950	4–5	Longitudinal	Parents completed a computer-assisted interview including questions regarding the type, fat content and frequency of milk intake.	Positive	Higher milk consumption by 4-year-olds was associated with higher z-scores of BMI and weight-for-height at 4 years. This corresponded to differences between children drinking <1 and ≥4 milk servings daily of approximately 0.15 kg in weight. By age 5 years only the association with height remained significant.
Vanderhout (2016) [76]	Canada (HIC)	2745	1–6	Cross-sectional	Parents answered a standardized data collection instrument adapted from the Canadian Community Health Survey	Inverse association between milk fat content and z-BMI score	A negative association between milk-fat percentage and zBMI. Participants who drank whole milk had a 5.4-nmol/L higher median 25(OH)D concentration and a 0.72 lower zBMI score than children who drank 1% milk. Whole milk consumption among healthy young children was associated with higher vitamin D stores and lower BM
Beck (2017) [77]	USA (HIC)	145	3	Cross-sectional	24-h-dietary recalls were conducted to determine child intake of whole, 2% or 1% milk in San Francisco based cohort of Latino children	Inverse association between high milk fat consumption and obesity	Severely obese children had a lower mean intake of milk fat (5.3 g vs. 8.9 g) and fewer drank any milk (79% versus 95% for not severely obese children). In the multivariate model, higher milk fat consumption was associated with lower odds of severe obesity. Higher milk fat consumption is associated with lower odds of severe obesity among Latino preschoolers.

^1^ Income category refers to classification according to World Bank 2017; ^2^ *N* = subjects treated/receiving intervention, where controls were included; BMI, body mass index.
